# Antimicrobial-Resistant Nontyphoidal *Salmonella* Infections, United States, 2004–2016

**DOI:** 10.3201/eid2710.211339

**Published:** 2021-10

**Authors:** Amrita Bharat, Colleen P. Murphy, Michael R. Mulvey, Saarah Hussain, Carolee A. Carson, Richard J. Reid-Smith, CIPARS Provincial Partnership

**Affiliations:** Public Health Agency of Canada, Winnipeg, Manitoba, Canada (A. Bharat, M.R. Mulvey);; Public Health Agency of Canada, Guelph, Ontario, Canada (C.P. Murphy, S. Hussain, C.A. Carson, R.J. Reid-Smith)

**Keywords:** *Salmonella*, nontyphoidal, antimicrobial resistance, antibiotic resistance, drug resistance, ceftriaxone, ciprofloxacin, ampicillin, incidence, resistance trends, foodborne diseases, bacteria, United States, enteric infections

**To the Editor:** Medalla et al. reported increased incidence of antimicrobial-resistant human infections with nontyphoidal *Salmonella* in the United States during 2004–2016 (*1*). When comparing incidence in 2004–2008 with that in 2015–2016, Bayesian hierarchical modeling estimated a 40% increase in the annual incidence of *Salmonella* infections with clinically important resistance (resistance to ampicillin or ceftriaxone or nonsusceptibility to ciprofloxacin). Most of the reported increases were attributed to serotypes I 4,[5],12:i:- and Enteritidis.

The US study used data from Laboratory-Based Enteric Disease Surveillance (https://www.cdc.gov/salmonella/reportspubs/surveillance.html) and the National Antimicrobial Resistance Monitoring System (https://www.cdc.gov/narms/index.html). The corresponding programs in Canada are the National Enteric Surveillance Program and the Canadian Integrated Program for Antimicrobial Resistance Surveillance (*2*,*3*). We used descriptive and univariable analyses without modeling for a preliminary comparison of data from these programs.

In Canada, yearly incidence (per 100,000 population) of human nontyphoidal *Salmonella* infections increased by 17% from 2004–2008 (median 18 cases) to 2015–2016 (median 21 cases). For nontyphoidal *Salmonella* (n = 20,665 isolates), resistance to ampicillin or ceftriaxone did not change substantially from 2004–2008 (ampicillin 15%; ceftriaxone 4%) to 2015–2016 (ampicillin 13%; ceftriaxone 5%). However, ciprofloxacin nonsusceptibility in nontyphoidal *Salmonella* increased from 7% in 2004–2008 to 15% in 2015–2016. For *Salmonella* Enteritidis (n = 6,694 isolates), resistance to ampicillin and ceftriaxone was uncommon (ampicillin 3%; ceftriaxone <1% for both 2004–2008 and 2015–2016). However, ciprofloxacin nonsusceptibility increased from 15% in 2004–2008 to 24% in 2015–2016. For *Salmonella* I 4, [5],12:i;- (n = 686 isolates), ampicillin resistance increased from 20% in 2004–2008 to 53% in 2015–2016, but ceftriaxone resistance decreased from 8% to 3%. Thus, increases were observed in both the United States and Canada for ciprofloxacin nonsusceptibility in *Salmonella* Enteritidis and for ampicillin resistance in *Salmonella* I 4, [5],12:i;-. Future modeling of surveillance data, enhanced by genomic analysis, will provide a more comprehensive comparison of findings for these countries.
